# IS VARIABILITY IN COGNITIVE PERFORMANCE ALWAYS MALADAPTIVE? THE MODERATING ROLE OF SOCIODEMOGRAPHIC FACTORS

**DOI:** 10.1093/geroni/igae098.3048

**Published:** 2024-12-31

**Authors:** Laura Klepacz, Eric Cerino, Matthew Pierce, Jeremy Hamm

**Affiliations:** North Dakota State University, Fargo, North Dakota, United States; Northern Arizona University, Flagstaff, Arizona, United States; North Dakota State University, Fargo, North Dakota, United States; North Dakota State University, Fargo, North Dakota, United States

## Abstract

Cognitive dispersion refers to an individual’s fluctuations in performance across a range of neuropsychological domains. Although past research has linked greater dispersion to cognitive decline in clinical populations, less is known about the characterization of dispersion in unimpaired, younger samples. The current study addresses this gap by identifying sociodemographic populations for whom dispersion may not be maladaptive and potentially linked to better cognitive performance over time. We pursued this aim by examining how age, sex, income, and education moderated the association between longitudinal changes in dispersion and corresponding shifts in cognitive performance using MIDUS data (n=2,518; age 55±11.2, Range: 33 – 83 years). Respondents completed a telephone-based battery of cognitive tests measuring several neuropsychological domains. Dispersion was calculated by generating an intraindividual standard deviation across 6 unique cognitive tasks. Results of OLS regression models showed that age (b=.01, SE=.00, p<.001), sex (b=.26, SE=.07, p<.001), income (b=-.01, SE=.01, p=.056), and education (b=-.02,SE=.01, p=.066) moderated the positive association between regressed change in dispersion and regressed change in mean performance. Specifically, increases in dispersion were most strongly associated with shallower 9-year declines in cognitive performance among comparatively older adults (b=.41, SE=.05, p<.001), women (b=.40, SE=.04, p<.001), individuals with less education (b=.35, SE=.05 p<.001), and those with lower income (b=.39, SE=.04, p<.001). Our study extends the literature by contributing to a more nuanced understanding of the populations for whom dispersion may not be maladaptive. These findings suggest that dispersion may reflect healthier cognitive aging in populations that are at greater risk for cognitive impairment.

